# The Complexity and Stigma of Pediatric Obesity

**DOI:** 10.1089/chi.2021.0003

**Published:** 2021-05-20

**Authors:** Andrea M. Haqq, Maryam Kebbe, Qiming Tan, Melania Manco, Ximena Ramos Salas

**Affiliations:** ^1^ Department of Pediatrics, Division of Pediatric Endocrinology, University of Alberta, Edmonton, Alberta, Canada.; ^2^ Department of Agricultural, Food and Nutritional Science, University of Alberta, Edmonton, Alberta, Canada.; ^3^ Pennington Biomedical Research Center, Baton Rouge, LA, USA.; ^4^ Unit for Multifactorial Diseases and Complex Phenotypes, Bambino Gesù Children's Hospital, Rome, Italy.; ^5^ Obesity Canada, University of Alberta, Edmonton, Alberta, Canada.

**Keywords:** genetic obesity, obesity stigma, pediatric obesity, stigmatization, weight bias, weight stigma

## Abstract

Weight stigma is rooted in a fundamental misunderstanding of the origins of obesity, wherein the interplay of behavioral, environmental, genetic, and metabolic factors is deemphasized. Instead, the widespread societal and cultural presence of weight stigma fosters misconceptions of obesity being solely a result of unhealthy personal choices. Weight stigma is pervasive in childhood and adolescence and can affect individuals throughout their life. Although the prevalence of pediatric obesity remains high throughout the world, it becomes increasingly important to understand how weight stigma affects weight and health outcomes in children and adolescents with overweight or obesity, including in those with rare genetic diseases of obesity. We identified and reviewed recent literature (primarily published since 2000) on weight stigma in the pediatric setting. Articles were identified with search terms including pediatric obesity, weight bias, weight stigma, weight-based teasing and bullying, and weight bias in health care. In this narrative review, we discuss the stigma of pediatric obesity as it relates to the complex etiology of obesity as well as describe best practices for avoiding bias and perpetuating stigma in the health care setting.

## Introduction

### Childhood Obesity

The high prevalence of childhood obesity is recognized as a global public health priority.^1^ It is estimated that obesity affects >107 million children worldwide, with the prevalence of pediatric obesity in high-income countries exceeding 20%.^2^ This represents a two- or threefold increase of obesity rates among children at the country-specific level within the past 40 years. In children and adolescents, the long-term health effects of obesity may last a lifetime and include obesity persisting into adulthood, increased risk for chronic diseases such as cardiovascular disease and type 2 diabetes, and early mortality.^2^

Immediate and long-term psychosocial health consequences, such as reduced self-esteem and depression, also arise in children with overweight and obesity.^3,4^ Of note, mental health concerns are the most commonly reported health risk among children with severe obesity,^5^ emphasizing the importance of incorporating psychological interventions into other aspects of obesity care. One such cause of poor physical health, mental health, and psychological outcomes in children with obesity is weight stigma.^4,6–8^

### Definition of Weight Stigma

Weight stigma is the attribution of negative beliefs (bias) based on weight, which can result in actions taken against the target of the bias (discrimination).^9,10^ This stigma can be directed toward children with overweight or obesity who do not fit social norms for body weight or shape.^11^ Stigma can arise from negative personal attitudes and views about obesity and individuals living with overweight or obesity and is often held by those with whom the individual interacts throughout childhood, including peers, educators, friends, family members, and health care providers.^10^ Actions involved in weight stigma include weight-based teasing; bullying; criticizing; harassing; victimization; and differential treatment by teachers, family, and friends, which can lead to social exclusion, marginalization, social inequities, and adverse health outcomes.^9,10,12^ An individual who is the target of weight stigma can internalize weight-biased beliefs and attitudes, where the awareness of the weight stigma can lead to agreement and application of the stereotypes to oneself, leading to self-devaluation.^13^

Stigma can stem from the notion that obesity is self-inflicted, wherein an individual does not adequately manage his or her weight through healthy eating and exercise.^10,14^ This belief oversimplifies weight management because it ignores biological and psychosocial factors that drive weight gain, many of which are beyond individual control.^14^ Another inaccurate belief surrounding weight management is that blaming individuals for their weight will provide motivation for weight loss and behavior change.^14^ In reality, shaming an individual for their weight does not prevent obesity; rather, it can have detrimental effects on weight management by promoting unhealthy coping strategies such as unhealthy eating behaviors (*e.g.,* binge eating) and avoidance of physical activity.^14,15^

To move beyond mindsets that promote weight bias, stigma, and discrimination, it may be necessary to evaluate transitioning away from models focused on the coupling of weight and health.^16^ The recognition of obesity as a chronic disease by health care organizations and professional societies has encouraged clinical and scientific communities to identify and understand the root causes of obesity.^1,17,18^ Nonetheless, stigma remains a pervasive issue in children and adolescents with obesity across multiple settings encountered in daily life. There remains a need to understand and appreciate the effects of stigma as a modifier of weight gain and poor health outcomes among children and adolescents with overweight and obesity due to various causes, including rare genetic diseases of obesity. The objective of this narrative review is to contextualize the stigma of pediatric obesity within its complex etiology, discuss the sources of weight stigma experienced by children and adolescents, give an overview of the psychosocial and physical consequences of weight stigma, and describe best practices for avoiding bias and perpetuating stigma in the health care setting.

## Methods

We performed a narrative review on weight stigma in the pediatric setting. In conceptualizing our narrative review, searching the literature, synthesizing data, and reporting results, we followed recommendations and standard subsections proposed by Green and colleagues for writing narrative literature reviews.^19^

A nonsystematic literature search was conducted using three electronic databases (PubMed, Medline, and Embase). Although no restraints were placed for publication date, we primarily chose to focus on literature published from January 2000 to February 2021 to review the most up-to-date literature. In addition to suggestions by the authors, reference lists from full-text articles that met our inclusion criteria were searched to identify any additional articles of relevance. Search terms included pediatric obesity, childhood obesity, rare genetic diseases of obesity, weight bias, weight stigma, weight-based discrimination, weight-based teasing, weight-based bullying, weight bias in health care, obesity stigma, obesity discrimination, and obesity causes in different combinations. Studies selected for inclusion in the review were focused on stigma in children and adolescents with obesity (*e.g.,* BMI ≥85th percentile), including long-term effects of weight stigma in childhood. Additional inclusion criteria included studies discussing the prevalence, consequences, and proposed interventions for weight stigma in settings relevant to pediatric individuals, including school, home, and health care facilities. Exclusion criteria included articles for which the full text was not available or articles that were not in English.

We prepared a brief synopsis of each article, identifying the study objectives, research design, and findings. For familiarization, authors read and reread the articles that met the inclusion criteria and took notes on each in a table of information. Information collected included the authors, year of publication, purpose of the study reviewed, study design, a review of the findings, conclusions, and mention of agreement or disagreement in the literature. After evaluation of the articles that met our inclusion criteria, results were organized into related areas as informed by the goals of the narrative review. These were then synthesized by the authors into five thematic sections to provide a meaningful overview of pediatric weight stigma. The five thematic sections are (1) prevalence and sources of pediatric weight stigma, (2) factors that contribute to pediatric weight stigma, (3) consequences of pediatric weight stigma, (4) pediatric weight stigma in the health care setting, and (5) interventions to reduce pediatric weight stigma.

## Prevalence and Sources of Pediatric Weight Stigma

### Prevalence of Weight Stigma

Weight stigma is pervasive, affecting children, adolescents, and adults with obesity, as well as parents and caregivers of children with obesity.^10,20^ The stigma of pediatric obesity has increased with the prevalence of obesity since the 1960s,^21^ but the profound effects of this stigma have not historically received the same level of attention as overt health-related consequences, such as cardiovascular disease, diabetes, and early mortality.^2,22^ However, recent studies have been investigating weight stigma in children. Children with obesity can experience weight-related bullying, teasing, and adverse outcomes including poor self-esteem, depressive disorders, impaired school performance, and minimal social involvement.^9,10,23,24^

**Figure 1.**
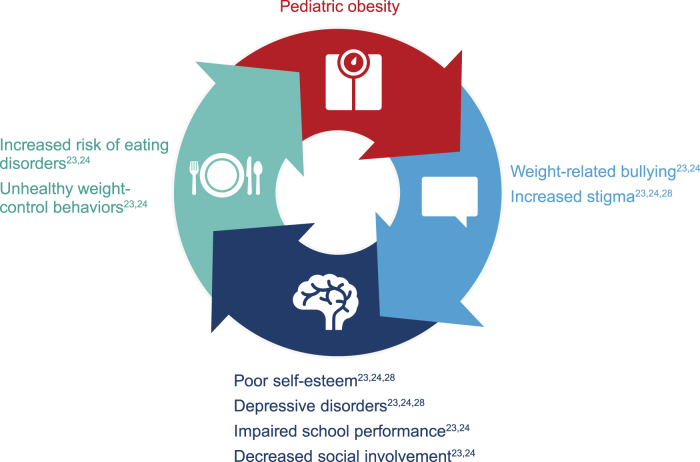
Negative feedback loop in pediatric obesity.

Indeed, in a study of students from 20 schools, 27% of the students reported weight-related teasing.^25^ The prevalence is higher among individuals who seek weight loss treatment, with one study finding that 64% of adolescents enrolled in a weight loss camp had experienced weight stigma, with 71% of these adolescents experiencing stigma at school in the last year.^26^ Weight stigma was also persistent, with ∼80% reporting stigma lasting >1 year and >33% reporting stigma lasting for ≥5 years. Importantly, children and adolescents with overweight or obesity are more vulnerable to weight bias and discrimination compared with their peers.^26,27^ In turn, psychosocial impairments can hinder weight management through unhealthy weight-control behaviors (*e.g.,* unhealthy diets), creating a negative feedback loop of stigma and weight gain (Fig. 1).^23,24,28^

### Sources of Weight Stigma

Children and adolescents with obesity experience stigma from multiple sources, including peers, educators, parents, media and entertainment, and health care providers.^9,24^ However, much of what is known about weight bias, discrimination, and stigma in children and adolescents comes from research primarily conducted in relation to the school setting. A meta-analysis indicated that children with overweight or obesity are 19% and 51% more likely, respectively, to be bullied by their peers than children without overweight or obesity.^27^ Furthermore, 41% of high school students who have observed stigmatization of their peers reported that having overweight was the primary reason for why an individual would be bullied.^12^ Weight stigma among adolescents most commonly took the form of teasing, ignoring, avoiding, excluding from social activities, and negative rumors, as well as verbal threats and physical harassment.^12^

## Factors That Contribute to Pediatric Weight Stigma

### Lack of Understanding of the Factors Influencing Obesity

**Figure 2.**
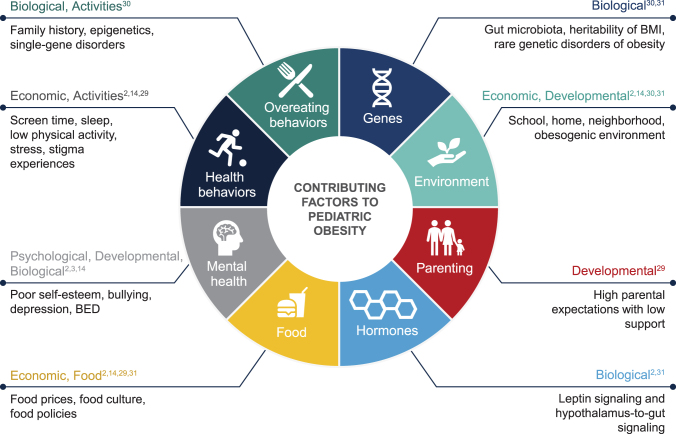
Factors contributing to pediatric obesity. BED, binge eating disorder.

Weight stigma is driven by the common disregard of the complex etiology of obesity. Pediatric obesity is a multifactorial chronic disease with a variety of phenotypes, clinical presentations, and treatment responses^17^ that result from the interplay of behavioral, environmental, genetic, and metabolic factors (Fig. 2).^2,3,14,29–31^ However, many hold the view that weight is within the control of an individual and incorrectly attribute overweight or obesity to individual failure in willpower or responsibility.^32^

The current public health narrative of obesity focuses mostly on the behavioral and environmental factors that have contributed to the rise in obesity prevalence worldwide.^15^ Such factors include technological advances that allow for a sedentary lifestyle, thereby decreasing energy expenditure, as well as increased availability and relative affordability of energy-rich foods (often served in large portions) and marketing of unhealthy food options, of which children are a prime target.^14,31^ Physiologically, however, incremental and sustained increases in body weight can reset energy homeostasis to a higher body weight set point and challenge weight management.^33^ The physiological and biological aspects of obesity are rarely part of obesity prevention and management narratives.

Despite the increasing prevalence of obesity worldwide, susceptibility to weight gain varies among individuals, suggesting there is a heritable component of overweight and obesity that interacts with such environments.^31^ Therefore, important in the obesity prevention and management discussion are the genetic components of the disease that are unrelated to the choices of the child or caregiver.^14^ Most cases of obesity are polygenic in nature, with multiple genes making small contributions to the overall phenotype. In this scenario, common gene variants associated with body weight are comparatively frequent within the population but have a modest effect on body weight.^31^

In contrast, genetic variants that have a pronounced effect on body weight are rare, yet notable by the extreme nature of the resulting phenotype, often presenting as early-onset obesity and severe obesity. Several forms of genetic obesity have been identified in humans, predominantly caused by variants of genes within the leptin–melanocortin pathway, which is involved in hypothalamic regulation of energy homeostasis.^30^ Common features of many rare genetic forms of obesity include early weight gain, often within the first or second year of life, and hyperphagia accompanied by insatiable hunger and preoccupation with food.^34^ However, there may be limited awareness and understanding of rare genetic diseases of obesity among health care professionals, policy makers, and the public, which could contribute to stigma and impact access to interventions and support for children and their families.

### Understanding Disordered Eating and Genetic Obesity Diseases: Moving Beyond Simplistic Obesity Solutions

**Figure 3.**
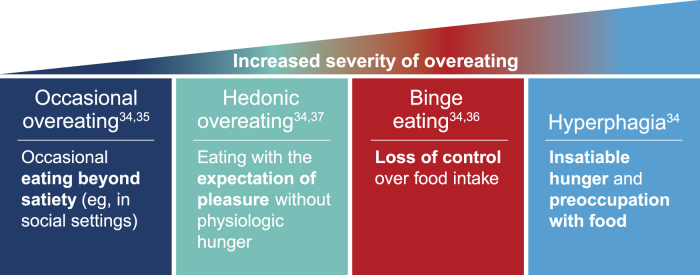
Spectrum of overeating behaviors.

Overeating behaviors exist on a spectrum ranging from occasional indulgence associated with special occasions or celebrations (overeating) to insatiable hunger (hyperphagia; Fig. 3).^34–37^ Whereas infrequent overeating is not restricted to those with overweight and obesity, severe disturbances in eating behaviors are associated with loss of control over consumption or underlying clinical or genetic conditions.^35^ Between these extremes are behaviors such as hedonic overeating and binge eating. Hedonic overeating is characterized by overeating despite lack of hunger and is driven by reward pathways that override inhibitory and homeostatic signals.^34,37^ Binge eating is another type of overeating behavior marked by recurrent episodes (≥1 per week for ≥3 months) of consuming a large amount of food, accompanied by feelings of loss of control and distress.^36^ Although binge eating disorder is common among individuals with obesity, it occurs among children with varying weight statuses, and obesity is not a requirement for a binge eating disorder diagnosis.^36^

Notably, overeating behaviors in childhood increase the risk of obesity and may influence the development of disordered eating. For instance, a study of 158 adolescent girls found that eating in the absence of hunger in childhood at age 7 years predicted binge eating behaviors at age 15 years.^38^ Furthermore, children with obesity have reported higher rates of loss-of-control eating and binge eating than children without obesity.^29^ Even the severity of childhood obesity may be associated with overeating or disordered eating behaviors. A study of 817 children with obesity (BMI >90th percentile) found that those who feel embarrassed or inhibited when eating in front of others were more likely to have severe obesity (defined as ≥120th percent of the 95th percentile of established growth curves) than those who did not.^39^ In addition, children with severe obesity were more likely to eat in the absence of hunger than those without severe obesity.^39^ Taken together, children with both obesity and disordered eating behaviors are at increased risk of obesity in adulthood or future eating disorders.^29^

An underlying genetic cause of overeating behaviors often presents distinctly and more severely from nongenetic forms of overeating. Genetic obesity, often associated with early-onset obesity and insatiable hunger, presents a unique case study for examining the pervasiveness of stigma and its effects in a population wherein weight gain is associated with a direct genetic cause. The most severe overeating behavior is hyperphagia, which is an overwhelming relentless hunger.^34^ Hyperphagia is associated with either physical alterations to the hypothalamus (*e.g.,* lesions or tumors) or genetic variants in hypothalamic leptin–melanocortin signaling pathways (*e.g.,* rare genetic diseases of obesity).^34,40^ Some rare variants in these pathways, such as those in the melanocortin 4 receptor, may account for 2%–3% of obesity in children and adults.^41^ Hyperphagia associated with variants in the leptin–melanocortin signaling pathway contributes to weight gain through impaired satiety and is characterized by a severe preoccupation with food (hyperphagic drive), which can lead to persistent, potentially extreme, or dangerous food-seeking behaviors (night eating, stealing food, and indiscriminate eating).^34^

Hyperphagic behaviors can present on a continuum of severity based on the underlying genetic cause; whereas hyperphagia can be less severe in individuals deficient for the melanocortin 4 receptor, individuals with Prader–Willi syndrome often exhibit aggressive food-seeking behaviors, indiscriminate eating (eating of nonfood items), and binge eating to the point of life-threatening stomach rupture or gastric necrosis.^34,42^ When denied food, individuals with hyperphagia experience significant distress. Hyperphagia is a significant source of stress not only for those experiencing it, but also for families, caregivers, and support systems, and requires constant vigilance to monitor relentless food-seeking behaviors.^34^ Caregivers of children with hyperphagia may need to lock up food to prevent binge eating or modify daily routines to manage their child's behavior or distract their child from food.^34^ In children with early-onset severe obesity, the presence of hyperphagia should alert health care providers to refer patients to a geneticist for evaluation of a potential underlying genetic cause of obesity.^40^ There are a number of scales that can assess hunger and hyperphagia symptoms, but there is a lack of standardized objective assessments for evaluating hyperphagic behaviors and symptoms.^34^ More awareness and understanding of the root causes of obesity in children, including overeating behaviors, is essential for reducing stigma and supporting children and their families who are affected by genetic diseases of obesity.^9^

## Consequences of Pediatric Weight Stigma

### Psychosocial Consequences of Weight Stigma

A robust and growing body of evidence supports a connection between stigmatization of children and adolescents with obesity and detrimental short- and long-term psychological and emotional effects affecting both individuals and their parents or caregivers that include psychosocial impairment, decreased executive function, reduced health-related quality of life, unhealthy weight control behaviors, and impaired weight management (Table 1).^3,4,6,8,20,43–45^ Studies have demonstrated that these negative effects can occur regardless of the source of stigma, which can include family, peers, educators health care providers, the media, and strangers.^3,4,9,20^ In a prospective longitudinal study of 5128 middle school children, approximately one-third reported at least one incident of perceived weight-based peer discrimination.^45^ Children who reported weight-based discrimination were more likely to experience body dissatisfaction, social anxiety, loneliness, and somatic symptoms (for girls but not boys). This marginalized position within society can result in social and academic inequities for adolescents living with obesity, such as being excluded from social networks, having fewer friendships than their peers, and having reduced performance or participation in school.^22,46^

**Table 1.** Consequences of Stigmatizing Obesity in Childhood and Adolescence

Increased risk for unhealthy weight control behaviors and binge eating^3,4^
Psychosocial impairment (depression, poor self-esteem, loneliness, and social anxiety)^3,4,45^
Parent and caregiver distress^20^
Additional weight gain^8^
Inability to maintain weight loss^44^
Reduced health-related quality of life^6^
Decrease in working memory^43^

Weight-based teasing is a common experience for children with obesity or overweight and can have adverse effects on psychological outcomes. Depression, anxiety, anger, and decreased self-esteem among children who experience weight-based teasing by both peers and family members are associated with the frequency of teasing events as well as the number of sources and the extent to which the child finds teasing bothersome.^3,4^ Adolescents who report a higher degree of distress from weight-based teasing demonstrate more depressive symptoms and lower self-esteem than those who are less distressed by teasing experiences.^47^

Among children with overweight, the severity of weight-based teasing is significantly associated with weight concerns, loneliness, negative personal perception of physical appearance, and strong preference for sedentary/isolative activities because of a fear of teasing. In addition, a study of 111 children aged 9–18 years found that higher reported internalized weight bias was significantly associated with more teasing from peers and lower self-esteem.^48^ Notably, internalized weight bias levels among children in the study were not associated with BMI *z* score or weight category, suggesting that bias internalization is independent of an individual's weight. The psychosocial impact of weight bias can also stem from the type of language parents use with their children. When surveyed, a group of adolescents seeking weight loss treatment expressed having negative emotional reactions, including feeling sadness, embarrassment, or shame, when their parents used weight-based language, including the terms “fat,” “high BMI,” or “big.”^49^ A study of 20 middle schools and high schools including 2793 students found that observing weight-related teasing at school was associated with increased depressive symptoms, body fat dissatisfaction, body build dissatisfaction, dieting, and decreased self-esteem in girls, as well as greater depressive symptoms in boys.^25^ Moreover, a study of 4086 students across 26 schools found that boys, regardless of their own weight, reported lower school belonging within schools that had high incidence of students being targeted based on weight.^50^ Furthermore, greater weight bias internalization by parents has been associated with a higher frequency of having child-centered conversations about weight.^51^

Pediatric weight stigma has also been found to negatively affect parents and caregivers. Parents report feelings of isolation, a sense of blame for their child's weight struggles, and fear regarding their child's health.^20,52^ Parents who themselves have experienced weight issues have a tendency to internalize self-blame for their child's weight.^53^ In a case study of a child with a rare genetic disease of obesity, the child's mother shared her experience, saying that “…many people told me I was a bad parent by letting her become so obese,” and that she began to “avoid [taking] her outside to prevent the emotional burden of the remarks people made.”^20^ Parents may also feel pressured to cook healthy home-made meals for their children.^54^ However, parents often face financial and temporal challenges when attempting to prepare these meals, leaving them feeling “frustrated and inadequate.”^54^ Therefore, it is crucial to consider the consequences of weight stigma holistically, taking both the pediatric and parental experience into consideration.

Overeating behaviors and weight stigma are deeply intertwined. Weight-based teasing has been linked to unhealthy weight control behaviors, binge eating, and disordered eating-related thoughts among children and adolescents.^3,4^ In a study of 80 children with obesity, those who experienced weight-based teasing from other children had five times greater risk for engaging in unhealthy weight control behaviors than children who were not teased.^4^ Maladaptive eating behaviors, including binge eating and eating as a coping mechanism, have also been associated with internalization of weight bias among adolescents seeking weight management treatment.^55^ On the “extreme” end of overeating, parents of children with hyperphagia experience shame and stigma associated with their child's obesity, including hurtful remarks from others about their child's weight and suspicion of child neglect.^20^ Diagnoses by health care providers may ease the impact of stigmatization for parents. Similar effects of weight stigma have been observed in sexual and gender minority youth, where high frequencies of weight-based victimization from family or peers were associated with increased maladaptive eating, dieting, and poor weight-related health outcomes.^56^ Taken together, it is crucial for health care providers to understand the various levels of overeating in children and adolescents with obesity and their associations with weight stigma.

Although worsened health outcomes can occur because of increased weight associated with weight stigma, some negative outcomes have been associated with the stigma itself, regardless of weight. A study of health-related quality of life in 600 children aged 8–11 years showed that experiencing stigma was a mediator for outcomes across all levels of body weight.^6^ In the best-fitting model, stigma experiences were responsible for the reduction of health-related quality of life, which occurred with increasing body weight in children. In a separate analysis of 176 children (average age, 9.7 years), a decreasing working memory was associated with increasing body weight when there was a threat of experiencing weight-based stereotypes (*i.e.,* that obesity is associated with lower intelligence).^43^ Together, these studies highlight the negative impact of weight stigma on physical health and executive function.

### Physical Health Consequences of Weight Stigma

Multiple studies have demonstrated that weight-based teasing among children and adolescents is associated with weight gain and impairs long-term weight loss maintenance.^7,8,44^ The longitudinal National Heart, Lung, and Blood Institute Growth and Health Study followed 2379 girls aged 10–19 years to assess the impact of being labeled “too fat” on obesity status.^57^ After adjusting for factors including baseline BMI, experiencing weight-based labeling by family and nonfamily members at age 10 years significantly predicted obesity at age 19 years. In an exploratory analysis of a longitudinal observational study that enrolled 110 children who have and are at risk for overweight/obesity because of elevated BMI or family history (*i.e.,* two parents with overweight or obesity), children with overweight more often reported occurrences of weight-based teasing than those without overweight, and experiencing weight-based teasing was associated with increases in BMI and fat mass over time compared with not experiencing weight-based teasing.^8^ A longitudinal cohort study of 1830 adolescents assessed weight-related outcomes over a 15-year interval.^7^ After adjustment for confounding variables (*e.g.,* demographic covariates and baseline BMI), a correlation was found between weight-based teasing in adolescence and both high BMI and obesity in adulthood. This association was found with both peer- and family-based teasing for women, but only for peer-based teasing in men. The observation that coping responses and weight stigma in adolescents vary by the source (*i.e.,* friends, peers, or family) has been reported by other investigators; for example, weight stigma from peers and friends was associated with more coping through avoidance behaviors than with weight stigma from family.^58^ A recent meta-analysis found a significant association between weight stigma and overweight/obesity, with a higher risk of overweight and obesity among young children compared with adolescents and among boys compared with girls.^59^ Furthermore, this meta-analysis demonstrated that weight stigma and increased BMI were predictive of each other in longitudinal studies.

Weight-based teasing was also predictive of future unhealthy eating behaviors such as binge eating, unhealthy weight control, and eating as a coping mechanism.^7^ A link was also found between childhood weight-related teasing and emotional eating. In a cohort of 2036 girls aged 14–19 years from the National Heart, Lung, and Blood Institute Growth and Health Study, adolescents who experienced being labeled “too fat” at age 14 years were more likely to report greater unhealthy weight control behaviors including bulimic tendencies, as well as a drive for thinness and body dissatisfaction, at age 19 years.^60^ In addition to unhealthy eating behaviors, weight stigma in adolescence may influence other unhealthy and dangerous behaviors over time. A 10-year longitudinal study of 1147 adolescent girls with unhealthy weight control behaviors found that experiencing harmful weight stigma in adolescence was predictive of future substance use.^61^ These findings highlight the persistent nature of weight-based discrimination and stigma occurring during childhood and adolescence. Indeed, in a questionnaire-based study of 299 female undergraduates, a history of overweight during childhood predicted lifetime weight stigma.^62^

Weight stigma may contribute to chronic disease through increased activation of physiologic pathways, including those involved with metabolism and inflammation. This may lead to elevated blood pressure, inflammatory markers, lipid levels, and glucose levels.^8,11^ Although in an analysis of adolescents with a broad weight distribution not seeking treatment and adolescents with obesity seeking weight loss treatment, weight stigma was not broadly associated with a worsened metabolic state or inflammation.^8^ In a study of 47 women with overweight or obesity, both the frequency of experienced weight stigma and the consciousness of the stigma were associated with oxidative stress and cortisol levels.^63^ Although abdominal adiposity was associated with cortisol and oxidative stress, the levels associated with weight stigma were above those that could be accounted for by abdominal or total adiposity. These findings support weight stigma as a factor that may contribute to the pathophysiology of obesity. This study, however, was conducted in adult women, and further research is necessary to understand whether the biological impact of weight stigma is similar in children and adolescents.

## Pediatric Weight Stigma in the Health Care Setting

The health care setting is a key place where children with obesity may encounter stigma. Weight stigma in health care settings can have serious implications on the quality of care that children and adolescents receive.^64^ The bulk of research on weight stigma in health care settings has involved adults with obesity, and there is limited research from the pediatric health care setting.^65,66^ A survey of 308 pediatric nurses and clinical support staff confirmed the presence of weight bias attitudes regarding the characteristics and perceived controllable behaviors of children with obesity.^65^ The use of stigmatizing language by health care providers can promote unhealthy behaviors and should be avoided.^67^ Indeed, when a cohort of 148 adolescents was asked to assess preferred language used by health care providers when discussing weight, terms including “extremely obese,” “obese,” and “fat” were rated low compared with terms including “weight problem,” “plus size,” and “BMI.”^68^ Notably, adolescents with higher levels of weight stigma internalization also reported differential language preference compared with those with lower internalization, showing significantly less preference for words including “large,” “curvy,” and “big” while adolescents with low levels of internalization preferred terms such as “heavy.”

Experiencing stigma from health care providers can also affect the health care decisions of parents of children with obesity. In a survey of 427 parents, 35% reported that perceived weight stigmatization from their child's doctor would lead them to seek a new health care provider, and 24% reported they would avoid future appointments.^69^ The overarching outcome of these experiences may lead to delaying or avoiding seeking care by children with obesity and their families. This can have serious implications for their health outcomes. Health care providers should avoid using stigmatizing language and practices and strive to make their clinical practices safe and accessible to their patients.^67^

## Interventions to Reduce Pediatric Weight Stigma

Current data clearly illustrate that weight stigma has both short- and long-term negative health and social effects on children and adolescents. As such, it is necessary to identify how best to eliminate weight bias and stigma, particularly in health care settings. Because weight bias is pervasive, there is a need to correct misperceptions about obesity at the population level. Efforts from other groups who have led stigma reduction campaigns can be leveraged.^16^ For instance, there is a role for patient activism in weight stigma reduction efforts. There are many patient organizations and initiatives around the world that are actively working to eliminate weight bias and improving lives of those with obesity through research, education, and action, including the Obesity Action Coalition (United States), Obesity Canada, the European Coalition for Persons Living with Obesity, the Foundation for Prader–Willi Research, and the Prader–Willi Syndrome Association (United States).

Building understanding about the complex causes of obesity and creating empathy through personal narratives may help to reduce biased beliefs about obesity. Narratives of personal experience from children and adolescents who face weight bias and discrimination can be powerful, but further stigma may occur for those who share these narratives.^16^ Another means of countering negative beliefs about obesity is to reframe obesity as a disease, which increases positive perceptions of people with obesity and thus reduces weight bias.^70^ In its 2018 position statement, The Obesity Society advocated shifting the public narrative of obesity from a lifestyle choice or behavioral issue to a chronic disease with many different causes.^17^ This is consistent with professional society and governmental policies in countries throughout the world that recognize obesity as a disease.^16^ In addition, the 2020 joint international consensus statement from a group of experts on obesity called for health care providers and other stakeholders to pledge to eliminate weight bias and stigma of obesity.^18^

From a research perspective, there is a need for understanding the causes of weight bias; identifying negative attitudes, beliefs, and behaviors that are modifiable; and developing and testing interventions aimed at reducing weight bias and its effects on children and adolescents with obesity.^71^ Interventions are needed at various levels (*e.g.,* obesity prevention and management interventions) and should be tailored to the audience and the various situations in which weight stigma is experienced (*e.g.,* at school, at home, in the media, and in the health care setting).^14,22^ Population level interventions are also needed. For example, social marketing and body diversity campaigns may be explored as interventions.^71^ There is also a need to understand how to improve communications between health care professionals and parents of children who have obesity. For example, CONversation Cards^©^ and Conversation Cards for Adolescents^©^ were developed to help parents or adolescents identify the barriers and enablers that they may encounter when addressing issues related to weight and health.^72,73^ However, more tools and resources are needed to help health care professionals develop nonjudgmental, nonweight biased therapeutic relationships with families and children.

### Avoiding Weight Bias in the Health Care Setting

**Figure 4.**
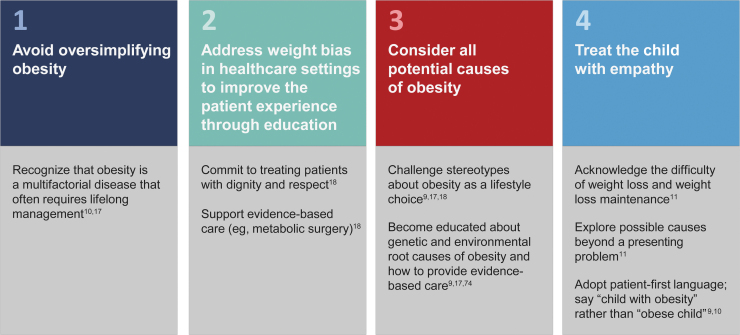
Health care provider best practices for avoiding stigmatization of obesity.

Patient perceptions of bias within the health care system contribute to weight stigma and adversely affect quality of care.^14,66^ From a patient interaction standpoint, there are several positive steps health care providers can take to avoid weight bias and perpetuation of the stigma of pediatric obesity (Fig. 4).^9,10,17,18,74^ At the core of their treatment model, health care providers should understand the complexity of obesity as a disease and the challenges facing long-term disease management.^9,18^ Health care providers should also avoid oversimplifying the causes of obesity (*e.g.*, suggesting patients “eat less fast food”). Of note, recognizing obesity as a disease by health care providers is associated with positive attitudes, no blaming, and empathy toward individuals with obesity.^70^

Furthermore, weight bias among health care providers has been observed across specialties and settings, including pediatric clinics.^9,66^ To improve patients' experiences, there is a fundamental need to change clinician-held beliefs and stereotypes regarding obesity that inform patient interactions. Nurses have indicated the importance of developing and maintaining trusting relationships with the parents of children with overweight or obesity.^75^ Education in the form of courses, online resources, and continuing education is needed to promote clinician awareness of weight stigma, convey the complex nature of the disease, and increase practitioner comfort with identifying and managing pediatric obesity.^74,76^ Obesity and weight stigma should also be included in curricula for medical professionals and health care providers, and providers specializing in treating obesity should provide evidence of their skills for stigma-free practice.^18,74^ Professional organizations should facilitate certification for health care provider knowledge of stigma and stigma-free practices.^18^ In addition, creating a clinic environment with the appropriate infrastructure and equipment that can accommodate patients of all sizes and implementing institutional policies to prevent weight bias may help provide comfortable and accessible health care for individuals with obesity.^18,76^

Health care providers must have empathy for patients with obesity; they should not blame patients or their parents for their condition and should instead focus on maintaining behavioral and health outcomes, given that even modest weight loss can significantly improve cardiovascular risk factors and diabetes.^10,16,77^ Another key recommendation widely endorsed by professional societies is the use of people-first language (*i.e.,* “patients with obesity” rather than “obese patients”).^10,16^ However, within the pediatric population, additional tailoring, such as using preferred body weight terms, may be appropriate.^68^ Future research should investigate whether people-first language is associated with reduction in weight bias. Altogether, there is an ongoing need to understand and identify the specific health care-related sources of weight stigma among the pediatric population and to develop strategies for mitigating the enactment of weight bias or discrimination in the health care setting.

In addition, in the pediatric health care setting, there is a unique opportunity to reduce stigma for children with rare genetic diseases of obesity and lessen the burden of their caregivers through early identification and diagnosis. Misunderstandings of the complex etiology of obesity or lack of awareness of the genetic pathogenesis of these rare diseases may contribute to the stigma experienced by these individuals. In addition, the onset of obesity often occurs early in life for individuals with rare genetic diseases of obesity, presumably leading to susceptibility for experiencing stigmatization beginning in early childhood.^34^ Because stigma can arise through a lack of understanding of the etiology of obesity, recognizing that weight gain and aberrant eating behaviors such as hyperphagia are the result of a disease process rather than behavioral and parental shortcomings can alleviate the stigma associated with early-onset obesity.^18,20^ Awareness and knowledge of rare genetic diseases of obesity may promote a broad understanding of the complex contributing factors to obesity and reduce weight stigma and facilitate a new scientifically grounded narrative of obesity.^18^ Moreover, a diagnosis allows for tailoring treatment approaches, including behavioral, psychological, and possible pharmacologic interventions. It is recommended that genetic testing be performed in individuals with early-onset severe obesity who have clinical features consistent with a genetic disease of obesity (particularly hyperphagia) and/or who have a family history of severe obesity.^40^

Optimizing care for children with rare genetic diseases of obesity would benefit from increased clinician awareness of both the disease and advanced diagnostic technologies. Lack of access to genetic screening panels for obesity and to tertiary care obesity centers is a limitation to early diagnosis and management at the health care system level. Little is currently known about the effect of weight stigma on care for children with rare genetic diseases of obesity, which should be the subject of future research.

## Conclusion and Future Directions

The stigma of pediatric obesity is a pervasive problem that exacerbates burdens already faced by children and adolescents with obesity and their parents/caregivers. Stigma contributes to psychosocial impairments, unhealthy eating behaviors, and weight gain, as well as an enduring negative influence on weight control that continues into adulthood. Despite the available evidence, substantial knowledge gaps remain. The current narrative of pediatric obesity is primarily focused on lifestyle with little emphasis on the biological drivers of weight gain, including genetics. Promoting a better understanding of the genetics of obesity and its complex etiology may promote reduced stigma, particularly among children and youth affected by genetic obesity diseases. Furthermore, the breadth of literature on weight stigma is centered on adults. Additional research on weight stigma, particularly in health care settings, and its psychological, physical, and biological consequences in a pediatric population is needed.

Although the health care community and policy makers better understand the negative effects of weight bias and discrimination, there has been a move toward recognizing and eliminating stigmatizing language and practices. Health care providers play an important role in minimizing the stigma associated with pediatric obesity through early identification and diagnosis of rare genetic diseases of obesity, recognition and elimination of personal biases, and positive interactions grounded in patient-centered care for children with obesity and their parents/caregivers. Systematic changes in health care, education, public policy, and the media are needed to change the narrative of obesity and promote stigma-free environments.
